# Maternal Diabetes Induces Immune Dysfunction in Autistic Offspring Through Oxidative Stress in Hematopoietic Stem Cells

**DOI:** 10.3389/fpsyt.2020.576367

**Published:** 2020-09-03

**Authors:** Jianping Lu, Meifang Xiao, Xiaoling Guo, Yujie Liang, Min Wang, Jianchang Xu, Liyan Liu, Zichen Wang, Gang Zeng, Kelly Liu, Ling Li, Paul Yao

**Affiliations:** ^1^ Department of Child Psychiatry, Kangning Hospital of Shenzhen, Shenzhen, China; ^2^ Hainan Women and Children’s Medical Center, Haikou, China; ^3^ Department of Pediatrics, Foshan Maternity and Child Health Care Hospital, Foshan, China

**Keywords:** autism spectrum disorders, cytokine, hematopoietic stem cells, oxidative stress, superoxide dismutase 2

## Abstract

Autism spectrum disorders (ASD) have been found to be associated with immune dysfunction and elevated cytokines, although the detailed mechanism remains unknown. In this study, we aim to investigate the potential mechanisms through a maternal diabetes-induced autistic mouse model. We found that maternal diabetes-induced autistic offspring have epigenetic changes on the superoxide dismutase 2 (SOD2) promoter with subsequent SOD2 suppression in both hematopoietic stem cells (HSC) and peripheral blood mononuclear cells (PBMC). Bone marrow transplantation of normal HSC to maternal diabetes-induced autistic offspring transferred epigenetic modifications to PBMC and significantly reversed SOD2 suppression and oxidative stress and elevated inflammatory cytokine levels. Further, *in vivo* human study showed that SOD2 mRNA expression from PBMC in the ASD group was reduced to ~12% compared to typically developing group, and the SOD2 mRNA level-based ROC (Receiver Operating Characteristic) curve shows a very high sensitivity and specificity for ASD patients. We conclude that maternal diabetes induces immune dysfunction in autistic offspring through SOD2 suppression and oxidative stress in HSC. SOD2 mRNA expression in PBMC may be a good biomarker for ASD diagnosis.

## Background

Autism spectrum disorders (ASD) are a group of neurodevelopmental disorders characterized by impairment of verbal communication and social skills in addition to restricted and repetitive behaviors. During the past few decades, the prevalence of ASD has significantly increased to a ratio of 1:59 in the United States (1–3). Many factors, including genetics/epigenetics, environmental risk factors, sex, and immune system (4), have been reported to contribute to ASD development, while the detailed mechanism remains largely unclear (3, 5–7).

We have recently reported that prenatal progestin exposure (6, 8, 9) and maternal diabetes (10–12) contribute to ASD development through suppressed expression of estrogen receptor β (ERβ) and superoxide dismutase 2 (SOD2) in neurons (12). Furthermore, our preliminary data showed that SOD2 expression in peripheral blood mononuclear cells (PBMC) was significantly decreased in the ASD group compared to the typically developing (TD) group. Since PBMC are typically derived from hematopoietic stem cells (HSC), we hypothesize that SOD2 suppression in PBMC is due to gene suppression of HSC during embryonic development (13, 14).

Multipotent HSC are located in the bone marrow (BM) niche and are responsible for the generation of blood and immune cells. Their origins can be tracked back to the embryo during cell differentiation and organogenesis (13, 14), and it has been reported that prenatal exposure to risk factors, such as progestins (15–17) and hyperglycemia (10–12, 18), can induce gene suppression through epigenetic changes in neurons, subsequently triggering ASD symptoms (8, 12). Thus, we hypothesize that related HSC that originate from the same affected embryo may experience gene suppression and similar epigenetic modifications. Subsequently, the PBMC may inherit similar modifications (19), triggering the dysfunction of immune cells (20, 21) and resulting in abnormal cytokine levels (22, 23).

In this study, we aim to investigate the potential mechanism for ASD-associated immune dysfunction and elevated cytokines (22, 24). Maternal diabetes-induced mouse offspring were established as the experimental autistic model (12), and we found that they showed significant autism-like behavior and neuronal SOD2 suppression compared to the control group. In addition, the autistic offspring were found to have epigenetic modifications on the SOD2 promoter with SOD2 suppression in both HSC and PBMC in addition to subsequent SOD2 suppression in PBMC. BM transplantation (BMT) of normal HSC to these autistic offspring significantly reversed SOD2 suppression and oxidative stress in PBMC and subsequent abnormal cytokines, indicating that SOD2 suppression and oxidative stress in PBMC is due to epigenetic inheritance from HSC. Further, *in vivo* human study showed that SOD2 mRNA expression in PBMC was reduced to ~15% in the ASD group compared to the TD group. We conclude that maternal diabetes induces immune dysfunction in autistic offspring through oxidative stress in HSC and that SOD2 mRNA levels in PBMC may be a good biomarker for the diagnosis of ASD patients.

## Materials and Methods

A detailed description can be found in Supplementary Information (see Data S1), and the related primers used in this study are shown in **Table S1**.

### *In Viv*o Mouse Experiments

#### Mouse Protocol 1: Generation of Autistic Offspring

Adult (3 months old) female mice were monitored for estrous cycles with daily vaginal smears. Only mice with at least two regular 4- to 5-day estrous cycles were included in the studies. Chronically diabetic female mice were induced through injection of 30mg/kg streptozocin (STZ, 0.05 M sodium citrate, pH 5.5) after an 8-h fasting period. Animals with blood glucose >250mg/dl were considered positive, while control (CTL) mice received only vehicle injections. The females were caged with proven males, and pregnancy was verified through observation of a sperm plug, which was designated as day 0 of pregnancy. The male offspring were separated from the dams on day 21 and fed until 7–8 weeks of age for further experiments. Some of the 7- to 8-week-old offspring were then used for autism-like behavior testing. The amygdala was isolated for mRNA analysis, and HSC cells were isolated from the tibia and femur while PBMC cells were isolated from the blood for gene expression and biomedical analysis.

#### Mouse Protocol 2: BMT of HSC

Male offspring from the CTL and STZ groups in Animal Protocol 1 were used as recipients for BMT. HSC were harvested from the tibias and femurs of the male offspring (4 months old) that were obtained from either the CTL or STZ group in Animal Protocol 1 as the donor for BMT. The isolated HSC were purified by density centrifugation using Histopaque 1083^®^ (#-1083-1, Sigma) and then resuspended in 10 ml of RPMI 1640 supplemented with 10% FBS and 2mM EDTA before being systemically transplanted (2 × 10^6^ cells) into the recipient male offspring (with CTL or STZ group) that had been lethally irradiated with 2 doses of 6 Gy 3 h apart (25). All transplant-recipient mice were set aside for a minimum of 4 weeks to allow for complete reconstitution of the BM (26) before they were then used for autism-like behavior analysis. PBMC were separated from the blood using Ficoll-Paque Plus lymphocyte separation medium (22), and were used for analysis of gene expression and inflammatory cytokine secretion along with isolated HSC. The experimental mice were randomly separated into four groups as follows: CTL mouse with BMT of HSC from CTL mouse (CTL/CTL-HSC); STZ mouse with BMT of HSC from CTL mouse (STZ/CTL-HSC); CTL mouse with BMT of HSC from STZ mouse (CTL/STZ-HSC); STZ mouse with BMT of HSC from CTL mouse (STZ/CTL-HSC).

### Immunostaining

The isolated PBMC were transferred to cover slips, and the cells were fixed in 4% paraformaldehyde for 20 min before being incubated with 0.3% Triton X-100 in PBS for 15 min. After blocking with 5% goat serum in PBS at room temperature for 30 min, cells were incubated with 8-oxo-dG anti-mouse antibody (# 4354-MC-050, from Novus Biologicals) for 12 h at 4°C and subsequently with secondary antibody Alexa Fluor 488. The cover slips were then mounted using antifade Mountant with DAPI (staining nuclei, in blue). The photographs were taken using a Confocal Laser Microscope (Leica, 20× lens) and quantitated by Image J. software.

### Analysis of Cytokines

Mouse cytokine secretions were obtained from PBMC supernatant, including IL-1β (Interleukin 1β), IL-6, and monocyte chemotactic protein-1 (MCP1), and were measured using Mouse IL-1β/IL-1F2 Quantikine ELISA Kit (#MLB00C), Mouse IL-6 Quantikine ELISA Kit (#M6000B), and Mouse CCL2/JE/MCP1 Quantikine ELISA Kit (#MJE00B), respectively, according to manufacturers’ instructions from R&D Systems (27).

### Human Study Protocol

The study of human subjects was approved by the Human Subjects Institutional Review Board from Hainan Women and Children’s Medical Center. Thirty-two cases of ASD children and 28 cases of matched TD children (2–6 years old) were identified and subjects participated in this study with informed written consent from their parents (9). ASD diagnosis was based on several clinical assessments by a multidisciplinary team and was further confirmed by licensed clinical psychologists and psychiatrists in Hainan Women and Children’s Medical Center using the DSM-5 (Diagnostic and Statistical Manual of Mental Disorders, Fifth Edition) as diagnostic criteria (9, 28, 29). Peripheral blood (3–5 ml) was withdrawn from the selected children and plasma was collected. Various cytokines, including IFNγ (type II interferon), IL-1α, IL-1Rα (Interleukin 1 receptor antagonist), IL-1β, IL-6, IL-8, MCP1, macrophage inflammatory protein-1α (MIP1α) and tumor necrosis factor-α (TNF-α), were measured using BIO-PLEX Pro™ Human Chemokine Panel (40-Plex #171AK99MR2) according to manufacturers’ instructions from BIO-RAD. Furthermore, PBMC were isolated from fresh blood using Lymphoprep™ reagents (#07861, from STEMCELL Technologies) for mRNA analysis of ERα, ERβ and SOD2. Combined PBMC from either the ASD or TD groups were used for protein analysis through western blotting. The ROC (Receiver Operating Characteristic) curve was established and the Pass/Fail Cutoff Value was defined based on SOD2 mRNA levels using SPSS 22 software for screening of ASD children.

### Statistical Analysis

The data was given as mean ± SEM, and all the experiments were performed at least in quadruplicate unless indicated otherwise. The unpaired Student’s t-tests or one-way analysis of variance (ANOVA) followed by the Turkey-Kramer test were used to determine statistical significance of different groups, and the two-way ANOVA followed by the Bonferroni *post hoc* test was used to determine the effect of social recognition. The ROC (Receiver Operating Characteristic) curve and Pass/Fail Cutoff Value was established using SPSS 22 software, and a *P* value of < 0.05 was considered significant (8, 30).

## Results

### Maternal Diabetes induces SOD2/ERβ Suppression in PBMC in Autistic Offspring

**Figure 1:**
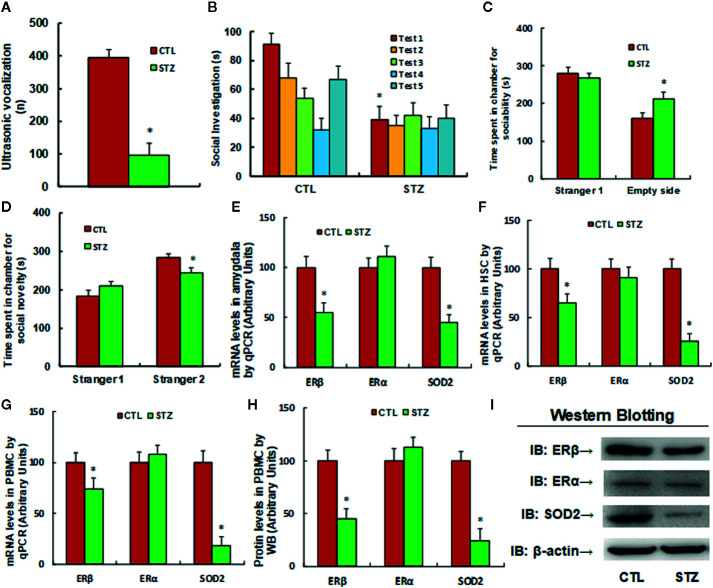
Maternal diabetes induces SOD2/ERβ suppression in PBMC in autistic offspring. The 6-week-old male offspring from either the control (CTL) or maternal diabetes (STZ) group were used for analysis. **(A–D)** Autism-like behavior analysis; **(A)** Ultrasonic vocalization, n = 9. **(B)** Social recognition, as indicated through the seconds spent socially investigating a conspecific [same conspecific in tests 1-4; novel conspecific in test 5 (a new stimulus mouse was introduced)], n = 9. **(C, D)** Three-chambered social tests, n = 8. **(C)** Time spent in chamber for sociability. **(D)** Time spent in chamber for social novelty. **(E)** mRNA levels in the amygdala, n = 5. **(F)** mRNA levels in HSC, n = 5. **(G)** mRNA levels in PBMC, n = 5. **(H)** The protein levels in PBMC, n = 5. **(I)** The representative pictures of western blotting for (H). *P < 0.05, vs. CTL group. Data were expressed as mean ± SEM.

The autistic mouse model was established using maternal diabetes-induced male offspring. We first evaluated autism-like behavior and found that ultrasonic vocalization frequency decreased to 24.5% in the diabetic (STZ) group compared to the control (CTL) group (see **Figure 1A**). Additionally, we conducted social recognition tests and found that there was a significant difference between the CTL and STZ groups [F (**1**,**16**) = 3.678, P = 0.017]. Subsequent *post hoc* analysis showed that habituation to the same stimulus conspecific (tests 1–4) was significant in the CTL group [F(3,32) = 4.793, P < 0.01] but not in the STZ group, and dishabituation was significant in the CTL group [F(1,8) = 3.961, P < 0.01] but not in the STZ group (see **Figure 1B**). We also evaluated these effects through three-chambered social tests. The results showed that time spent in the empty side of the chamber indicating sociability increased to 132% (see **Figure 1C**), while time spent in the empty side of the chamber indicating social novelty decreased to 86% (see **Figure 1D**) in the STZ group, compared to the CTL group. Our results confirm that maternal diabetes induces autism-like behavior in male offspring. Furthermore, we evaluated gene expression in the mice and found that mRNA levels of ERβ and SOD2 in the amygdala in the STZ group were decreased to 55% and 44%, respectively, compared to the CTL group, while ERα mRNA levels did not change (see **Figure 1E**). We then evaluated mRNA expression in HSC, and the results showed that ERβ and SOD2 mRNA levels in the STZ group decreased to 65% and 26%, respectively, compared to the CTL group, while ERα mRNA levels showed no significant changes (see **Figure 1F**). Finally, we evaluated gene expression in PBMC and found that ERβ and SOD2 mRNA levels in the STZ group decreased to 74% and 18%, respectively, compared to the CTL group (see **Figure 1G**). We also evaluated protein expression for these genes and observed a pattern similar to that of the mRNA levels, while ERα expression did not change (see **Figures 1H, I** and **Figure S1A**). Our results indicate that maternal diabetes induces SOD2/ERβ suppression in PBMC in autistic offspring.

### Transplantation of BM HSC Does Not Reverse Maternal Diabetes-Induced Autism-Like Behavior in Autistic Offspring

We evaluated the potential effect of BMT of HSC on the mice. The 6-week-old male offspring from either the control (CTL) or maternal diabetes (STZ) groups received HSC transplantation from either the control (CTL-HSC) or maternal diabetes (STZ-HSC) groups. The mice were used for autism-like behavior analysis 5 weeks after transplantation and the amygdala tissues or neurons were isolated for analysis. We first measured epigenetic changes on the SOD2 promoter in amygdala neurons, and the result showed that STZ group (STZ/STZ-HSC) significantly increased H3K9me2 modification on the SOD2 promoter to 267% compared to the CTL group (CTL/CTL-HSC), while HSC transplantation of either CTL-HSC (STZ/CTL-HSC group) or STZ-HSC (CTL/STZ-HSC group) had no effect (see **Figure S2A**), indicating that BMT of HSC cells does not affect the epigenetic changes in amygdala neurons. We then measured gene expression in the amygdala. The results showed that mRNA levels of ERβ and SOD2 significantly decreased in the STZ group compared to the CTL group, while ERα mRNA levels did not change, and HSC transplantation showed no effect on gene expression (see **Figure S2B**). We then evaluated autism-like behavior (ALB) in the mice and found that HSC transplantation had no effect on maternal diabetes-induced autism-like behavior, which included ultrasonic vocalization (see **Figure S2C**), social recognition tests (see **Figure S2D**) and three-chambered social tests (see **Figures S2E, F**). Our results indicate that HSC transplantation does not reverse maternal diabetes-induced autism-like behavior in offspring, which may be explained by the hypothesis that BMT cannot change epigenetic modifications on the SOD2 promoter, subsequently having no effect on the gene expression of ERβ/SOD2 in the amygdala.

### Transplantation of BM HSC Reverses Maternal Diabetes-Induced Gene Suppression in PBMC in Autistic Offspring

**Figure 2:**
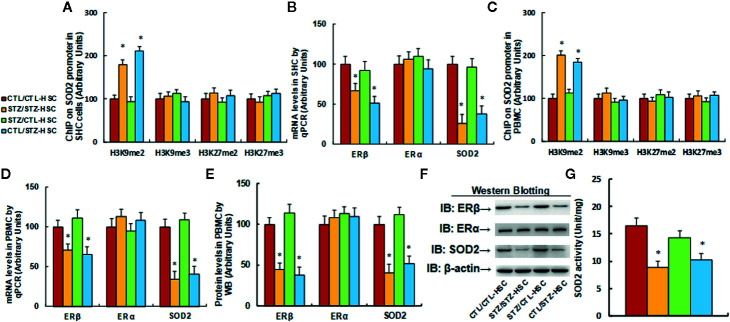
|Transplantation of bone marrow HSC reverses maternal diabetes-induced gene suppression in PBMC in autistic offspring. The 6-week-old male offspring from either the control (CTL) or maternal diabetes (STZ) group received transplantation of bone marrow HSC from either the control (CTL-HSC) or maternal diabetes (STZ-HSC) group, and mice were used for further biomedical analysis 5 weeks after transplantation. **(A)** The HSC were isolated for ChIP analysis, n = 4. **(B)** The mRNA levels in HSC, n = 4. **(C)** The PBMC were isolated for ChIP analysis, n = 4. **(D)** The mRNA levels in PBMC, n = 4. **(E)** The representative pictures of western blotting. **(F)** The quantitated protein levels in PBMC for **(E)**, n = 5. **(G)** SOD2 activity assay, n = 5. *P < 0.05, vs. CTL/CTL-HSC group. Data were expressed as mean ± SEM.

We first evaluated the effect of HSC transplantation on epigenetic changes on the SOD2 promoter in HSC. The results showed that in the maternal diabetes group (STZ/STZ-HSC), H3K9me2 modification increased to 179% compared to the control group (CTL/CTL-HSC) and transplantation of control HSC to the diabetic group (STZ/CTL-HSC) completely reversed this effect, while transplantation of diabetic HSC to the control group (CTL/STZ-HSC) mimicked the maternal diabetes-induced effect. On the other hand, there was no effect on the other type of histone methylation on the SOD2 promoter (see **Figure 2A**). Our results indicate that HSC transplantation from graft mice was successful for regeneration of HSC in host mice. We then measured mRNA expression in HSC and found that in the STZ/STZ-CTL group, mRNA levels of ERβ and SOD2 decreased to 67% and 51%, respectively, compared to the CTL/CTL-HSC group (see **Figure 2B**). We then evaluated the epigenetic changes in PBMC and found that in the STZ/STZ-HSC and CTL/STZ-HSC group, H3K9me2 modification increased to 201% and 184%, respectively, compared to the control group (CTL/CTL-HSC), while STZ/CTL-HSC totally normalized this effect. Additionally, there was no effect on the other type of histone methylation on the SOD2 promoter (see **Figure 2C**). Our results indicate that epigenetic changes were transferred from transplanted HSC to subsequent PBMC. We then measured mRNA expression in PBMC and found that in the STZ/STZ-CTL group, mRNA levels of ERβ and SOD2 were decreased to 71% and 65%, respectively, compared to the CTL/CTL-HSC group (see **Figure 2D**). We also measured the protein levels for those genes, and a pattern similar to that of the mRNA levels was observed (see **Figures 2E, F** and **Figure S1B**), while there was no significant effect on ERα expression. Finally, we evaluated SOD2 activity in PBMC. The results showed that in the STZ/STZ-CTL group, SOD2 activity decreased to 54% compared to the CTL/CTL-HSC group (seer **Figure 2G**). The STZ/CTL-HSC treatment completely reversed the maternal diabetes-induced effect for all the above measurements, while CTL/STZ-HSC treatment mimicked this effect. Our results indicate that HSC transplantation restores maternal diabetes-induced gene suppression in PBMC in offspring, which may be because transplanted HSC eventually differentiated into PBMC during subsequent immunological reconstitution after HSC transplantation.

### Transplantation of BM HSC Reverses Maternal Diabetes-Induced Oxidative Stress in PBMC in Autistic Offspring

**Figure 3:**
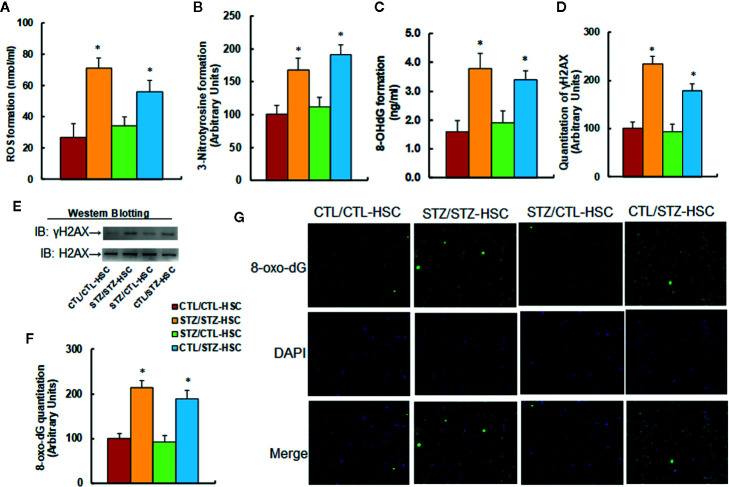
Transplantation of bone marrow HSC reverses maternal diabetes-induced oxidative stress in PBMC in autistic offspring. The 6-week-old male offspring from either the control (CTL) or maternal diabetes (STZ) groups received transplantation of bone marrow HSC from either the control (CTL-HSC) or maternal diabetes (STZ-HSC) group, and the mice were used for further biomedical analysis 5 weeks after transplantation. **(A)** ROS formation in PBMC, n = 5. **(B)** Quantitation of 3-nitrotyrosine formation, n = 5. **(C)** 8-OHdG formation, n = 5. **(D)** Quantitation of γH2AX formation. **(E)** Representative γH2AX western blotting band for **(D)**, n = 5. **(F)** Quantitation of 8-oxo-dG formation, n = 5. **(G)** Representative pictures of 8-oxo-dG staining for oxidative stress (green) and DAPI staining for nuclei (blue) in PBMC, n = 4. *P < 0.05, vs. CTL/CTL-HSC group. Data were expressed as mean ± SEM.

We evaluated the potential effect of HSC transplantation on oxidative stress in PBMC. We first measured reactive oxygen species (ROS) generation and found that STZ/STZ-CTL treatment increased ROS generation (see **Figure 3A**) and 3-nitrotyrosine (3-NT) formation (see **Figure 3B**) to 263% and 214%, respectively, compared to the CTL/CTL-HSC group. We then measured DNA damage and found that in the STZ/STZ-CTL group, 8-OHdG formation (see **Figure 3C**) and γH2AX formation (see **Figures 3D, E** and **Figure S1C**) increased to 238% and 234%, respectively, compared to the CTL/CTL-HSC group. We also evaluated 8-oxo-dG formation and found that 8-oxo-dG formation in the STZ/STZ-CTL group (see **Figures 3F, G**) increased to 214% compared to the CTL/CTL-HSC group. The maternal diabetes-induced effect was completely reversed in the STZ/CTL-HSC group for all the above measurements, while CTL/STZ-HSC group mimicked this effect. Our results indicate that HSC transplantation restores maternal diabetes-induced oxidative stress in PBMC in autistic offspring.

### Transplantation of BM HSC Reverses Maternal Diabetes-Induced Inflammatory Cytokine Release From PBMC in Autistic Offspring

**Figure 4:**
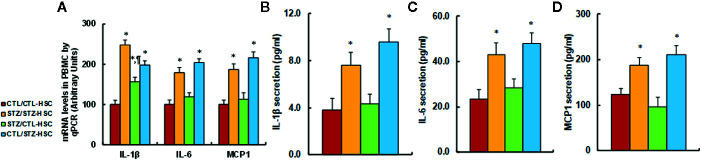
Transplantation of bone marrow HSC reverses maternal diabetes-induced inflammatory cytokine release from PBMC in autistic offspring. The 6-week-old male offspring from either the control (CTL) or maternal diabetes (STZ) group received transplants of bone marrow HSC from either the control (CTL-HSC) or maternal diabetes (STZ-HSC) group, and the PBMC were isolated for further analysis. **(A)** mRNA levels by qPCR, n = 4. **(B)** IL-1β secretion, n = 5. **(C)** IL-6 secretion, n = 5. **(D)** MCP1 secretion, n = 5. *P < 0.05, vs. CTL/CTL-HSC group; ¶*P* < 0.05, vs. STZ/CTL-HSC group. Data were expressed as mean ± SEM.

We evaluated the potential effect of HSC transplantation on inflammatory cytokine release in PBMC. We first evaluated mRNA levels for the cytokines and found that in the STZ/STZ-CTL group, mRNA levels of IL-1β, IL-6, and MCP1 increased to 248%, 179%, and 187%, respectively, compared to the CTL/CTL-HSC group, and the STZ/CTL-HSC group either partly (for IL-1β) or completely (for IL-6 and MCP1) reversed the maternal diabetes-induced effect, while CTL/STZ-HSC group mimicked this effect (see **Figure 4A**). We then evaluated the levels of cytokine proteins that were secreted from PBMC. The results showed that protein secretion of IL-1β (see **Figure 4B**), IL-6 (see **Figure 4C**) and MCP1 (see **Figure 4D**) in the STZ/STZ-CTL group increased to 197%, 184%, and 151%, respectively, compared to the CTL/CTL-HSC group, and STZ/CTL-HSC group completely reversed, while the CTL/STZ-HSC group mimicked, the maternal diabetes-induced effect. Our results indicate that HSC transplantation restores maternal diabetes-induced inflammatory cytokine release from PBMC in autistic offspring.

### Expression of SOD2 and ERβ in PBMC Decreased in ASD Patients

**Figure 5:**
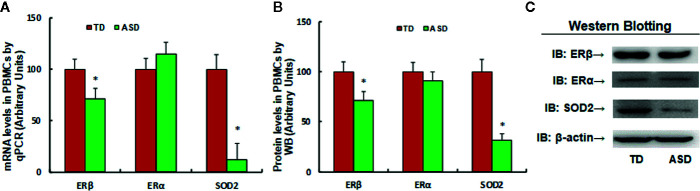
Expression of SOD2/ERβ in PBMC decreased in ASD patients. 3 ml of peripheral blood was withdrawn from either control (CTL, n = 61) or ASD (n = 64) children (2–6 years old), and the PBMC were isolated for mRNA analysis through real time PCR. *P < 0.05, vs. CTL group; ¶*P* < 0.01, vs. CTL group.

We found that SOD2/ERβ expression was suppressed in PBMC in maternal diabetes-induced autistic offspring in the mouse model. In order to verify whether similar suppression occurs in autistic children, 61 cases of TD and 64 cases of autistic (ASD) children were identified and the PBMC were isolated for gene expression analysis. The results showed that mRNA levels of ERβ and SOD2 in ASD group were decreased to 71.3% and 12.4%, respectively, compared to the TD group. We also measured the protein levels for those genes, and a pattern similar to that of mRNA levels was observed (see **Figures 5B, C** and **Figure S1D**). On the other hand, the ERα expression did not change (see **Figure 5**). Our results indicate that expression of SOD2 and ERβ in PBMC decreased in ASD patients, and SOD2 mRNA levels had the most significant decrease (~88%) in the ASD group compared to the TD group.

### Establishment of Pass/Fail Cut/Off Value for the Diagnosis of ASD Patients

**Figure 6:**
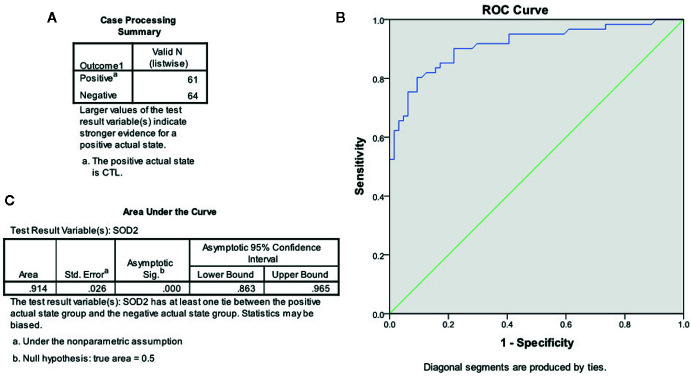
The ROC curve based on SOD2 expression for ASD diagnosis. The SOD2 mRNA expression levels from both CTL (n = 61) and ASD (n = 64) patients were used to draw the ROC (receiver operating characteristic) curve. **(A)** Case processing summary, positive represents CTL cases, and negative represents ASD cases. **(B)** ROC curve. **(C)** Area under the curve.

In order to establish the Pass/Fail Cut/Off value for the diagnosis of ASD patients, the ROC (Receiver Operating Characteristic) curve was established by SPSS 22 software using the original SOD2 mRNA expression levels (see **Figure 6**). Sixty-one cases of TD children (considered as positive) and 64 cases of ASD children (considered as negative) were used for calculations (see **Figure 6A**), and the ROC curve is shown in **Figure 6B**. Area Under the Curve was calculated to be 0.914 (see **Figure 6C**), showing very good sensitivity and specificity for ASD diagnosis in general. We then established the Pass/Fail Cut/Off value for the diagnosis of ASD patients using the coordinates of the curve. As shown in **Figure S3**, the Pass/Fail Cut/Off value was set as 0.0306 for SOD2 mRNA levels with 85% sensitivity and 83% specificity. We concluded that a value of <0.0306 in regards to SOD2 mRNA expression was considered to potentially indicate ASD in patients.

### ASD Patients Have Increased Levels of Inflammatory Cytokines in the Plasma

We evaluated the inflammatory cytokine levels in the plasma of both TD and ASD patients. Twenty-eight TD control cases and 32 ASD patient cases were selected, and the plasma were prepared to measure the cytokine levels. We found that the cytokine levels of IL-1α, IL-6, and MCP1 in the ASD group increased to 217%, 246%, and 154%, respectively, compared to the TD control group. On the other hand, there was no significant difference in the cytokine levels of IFNγ, IL-1Rα, IL-1β, IL-8, MIP1α, and TNF-α (see **Table 1**). Our results indicate that ASD patients have increased inflammatory cytokine levels compared to the TD group.

**Table 1:** Cytokine levels in plasma for typically developing (TD) and autistic (ASD) children.

Plasma cytokines	TD subjects (n = 28)Mean ± SDV	ASD subjects (n = 32)Mean ± SDV	*P* value
Age (years)	3.2 ± 0.8	3.1 ± 0.9	0.974
Male/Female	16:12	28:4	N/A
IFNγ (pg/ml)	8.61 ± 2.13	11.6 ± 3.11	0.087
IL-1α (pg/ml)	21.63 ± 8.61	47.04 ± 12.64	<0.01**
IL-1Rα (pg/ml)	87.44 ± 18.11	69.78 ± 21.56	0.416
IL-1β (pg/ml)	14.73 ± 3.56	15.9 ± 4.12	0.916
IL-6 (pg/ml)	12.76 ± 2.94	31.41 ± 5.67	<0.01**
IL-8 (pg/ml)	21.36 ± 4.57	23.65 ± 4.19	0.287
MCP1 (pg/ml)	36.91 ± 9.24	56.91 ± 10.23	0.036*
MIP1α (pg/ml)	14.23 ± 4.31	25.32 ± 6.27	<0.01
TNF-α (pg/ml)	15.64 ± 3.48	13.61 ± 2.22	0.798

ASD, autism spectrum disorders; TD, typically developing; * indicates P < 0.05; ** indicates P < 0.01.

## Discussion

In this study, we demonstrated that maternal diabetes-induced mouse autistic offspring have epigenetic modifications and SOD2 suppression in both HSC and PBMC. BMT of normal HSC to maternal diabetes-induced offspring reversed SOD2 suppression and elevated cytokine levels in PBMC. *In vivo* study further proved that ASD patients have significantly decreased SOD2 expression in PBMC. Our results indicate that immune dysfunction in ASD may be partly due to damage of HSC during embryonic development.

We have previously found that prenatal progestin exposure-induces autism-like behavior in offspring through ERβ/SOD2 suppression in neurons. Overexpression of ERβ in amygdala partly restores this effect, and male offspring are more susceptible than female offspring due to lower basal ERβ/SOD2 expression levels in neurons (8). Furthermore, we have recently showed that maternal diabetes induces autism-like behavior through epigenetic changes on the SOD2 promoter with subsequent SOD2 suppression in the amygdala (12). Our results indicate that expression of ERβ/SOD2 in the amygdala plays an important role in autism-like behavior. In this study, the maternal diabetes-induced autistic mouse model was established, and the male offspring was used for experiments to avoid potential interference from estrogen in female offspring. Additionally, around 50% of diabetic dams were either infertile or had born dead offspring; to avoid this, the dosage of STZ was reduced from 50 to 35 mg/kg to achieve mild diabetes in dams and subsequently increase the birth rate. We found that maternal diabetes-induced autistic offspring have epigenetic changes on the SOD2 promoter in amygdala neurons as well as in HSC and BPMC. Furthermore, HSC transplantation transferred the epigenetic changes to PBMC during HSC differentiation, restored the gene expression in PBMC, and subsequently restored maternal diabetes-induced oxidative stress and abnormal cytokine levels in PBMC. On the other hand, HSC transplantation showed no effect on amygdala neurons in terms of either epigenetic modifications or gene expression and subsequently showed no effect on maternal diabetes-induced autism-like behavior. Our results indicate that maternal diabetes-induced autism-like behavior is mainly due to prenatal damage of neurons (such as in the amygdala), instead of HSC or PBMC. It seems that immune dysfunction (triggered by dysfunction of PBMC and HSC) is not the root cause of ASD. On the other hand, the ASD-associated immune dysfunction may be at least partly due to parallel damage of HSC during prenatal exposure of risk factors such as hyperglycemia (12) or progestin (8). Our findings provide a new strategy for ASD clinical treatment. Restoration of either immune dysfunction in PBMC or physiological function (such as cytokine levels) may have little effect, while restoration of epigenetic changes in neurons may ameliorate autistic symptoms (6).

Many gestation insults and factors, including maternal infection, maternal immune activation, immunogenetics, and autoimmune disorders, have been reported to be associated with ASD development (21–24), while the detailed mechanism remains unknown. Multipotent HSC (31) are responsible for the generation of most adult blood and immune cells (14). Prenatal risk factors including progestins (15–17) and hyperglycemia (10–12, 18) may bring potential damage to HSC during embryonic development and subsequently affect the PBMC and related immune function. Our results show that maternal diabetes induces epigenetic changes on the SOD2 promoter in HSC and that these kinds of epigenetic changes are inherited in subsequent PBMC, resulting in immune dysfunction. BMT of normal HSC restores maternal diabetes-induced epigenetic changes in PBMC and subsequent PBMC dysfunction in autistic offspring, providing powerful evidence that immune dysfunction is at least partly due to HSC dysfunction triggered by prenatal spikes during embryonic development.

It has been reported that ASD is associated with elevated cytokine levels, although the detailed mechanism remains unclear (21–23, 32). In this study, we showed that maternal diabetes-induced autistic mouse offspring have elevated levels of inflammatory cytokines, including IL-1β, IL-6, and MCP1, and BM transplantation of normal HSC significantly restored abnormal cytokine levels to a normal amount. Furthermore, the human study showed significantly increased plasma cytokine levels of IL-1α, IL-6, and MCP1 in the ASD group compared to the TD group. Interestingly, SOD2 suppression in either HSC or BPMC is associated with ASD, indicating that SOD2 suppression and subsequent oxidative stress in HSC may potentially contribute to elevated cytokines in ASD (20, 33). It has been reported that nuclear factor-κB (NFκB) binding activity in PBMC increases significantly in ASD patients (34), and NFκB is an important mediator for the inflammatory process. Furthermore, hyperglycemia-mediated oxidative stress causes persistent NFκB activation through epigenetic changes (35). With this in mind, we suggest that SOD2 suppression in HSC may trigger elevated cytokine release through oxidative stress-mediated NFκB activation.

We have previously reported that maternal diabetes induces epigenetic modification on the SOD2 promoter with subsequent SOD2 suppression and oxidative stress in neurons, triggering ASD development in offspring (12). In addition, prenatal progestin exposure triggers SOD2 suppression in neurons in addition to ASD development (15–17). Here, we found that similar SOD2 suppression occurred in both HSC and PBMC in maternal diabetes-induced autistic offspring due to inheritance of epigenetic changes on the SOD2 promoter. We then hypothesize that SOD2 suppression in PBMC is associated with ASD. Our further *in vivo* study showed that SOD2 mRNA expression was reduced to ~12% in the ASD group compared to the TD group, this is a very significant dramatic reduction in SOD2 expression, indicating that maternal diabetes and prenatal progestin exposure may play a dominant role for the contribution of ASD development. Furthermore, the SOD2 mRNA level-based ROC curve shows very high sensitivity and specificity for ASD diagnosis.

### Conclusions

This study has determined that maternal diabetes-induced mouse autistic offspring have epigenetic changes on the SOD2 promoter that result in SOD2 suppression in both HSC and PBMC. BM transplantation of normal HSC reverses epigenetic changes and subsequently normalizes SOD2 suppression and elevated cytokine levels in PBMC. We conclude that maternal diabetes induces immune dysfunction in autistic offspring through oxidative stress in HSC (36) and that SOD2 suppression in PBMC can be a sensitive biomarker for ASD diagnosis.

## Data Availability Statement

The datasets presented in this study can be found in online repositories. The names of the repository/repositories and accession number(s) can be found in the article/**Supplementary Material**.

## Ethics Statement

The studies involving human participants were reviewed and approved by Hainan Women and Children’s Medical Center. Written informed consent to participate in this study was provided by the participants’ legal guardian/next of kin. The animal study was reviewed and approved by Institutional Animal Care and Use Committee from Kangning Hospital of Shenzhen.

## Author Contributions

PY wrote the paper. PY, LL, and JL designed, analyzed the data, and interpreted the experiments. XG, YL, JX, and ZW performed statistical analysis and part of the mouse experiments. MW, LL, GZ, and KL performed part of the gene analysis. JL and MX performed the remaining experiments. All authors contributed to the article and approved the submitted version.

## Funding

This study was supported by Shenzhen Science and Technology Innovation Committee Project #: JCYJ20160429185235132; Sanming Project of Medicine in Shenzhen #: SZSM201612079; Shenzhen Key Medical Discipline Construction Fund #: SZXK042; Shenzhen Double Chain Grant #: [2018]256 & Bureau of Public Health of Hainan Province Key Project #: 14A110065.

## Conflict of Interest

The authors declare that the research was conducted in the absence of any commercial or financial relationships that could be construed as a potential conflict of interest.

## Abbreviations

ALB, autism-like behavior; ASD, autism spectrum disorders; BMT, bone marrow transplantation; ChIP, chromatin immunoprecipitation; HO1, heme oxygenase 1; HSC, hematopoietic stem cells; PBMC, peripheral blood mononuclear cells; IL-6, interleukin-6; IL1β, interleukin-1β; IFNγ, type II interferon; MCP1, monocyte chemoattractant protein-1; MIP1α, macrophage inflammatory protein-1α; NFκB, nuclear factor-κB; ROS, reactive oxygen species; SOD2, superoxide dismutase 2; STZ, streptozotocin. TD, typically-developing; TNF-α, tumor necrosis factor-α.
