# Supplementary material for: Maternal Diabetes Induces Immune Dysfunction in Autistic Offspring Through Oxidative Stress in Hematopoietic Stem Cells
Source: Front Psychiatry. 2020 Sep 3;11:576367. doi: 10.3389/fpsyt.2020.576367 (PMC7495463; doi:10.3389/fpsyt.2020.576367)
Supplement: Supplementary file 1 [file Table_1.docx]

**Maternal Diabetes Induces Immune Dysfunction in Autistic Offspring through Oxidative Stress in Hematopoietic Stem Cells**

Jianping Lu^1,^*^,#^, Meifang Xiao^2,^*, Xiaoling Guo^3^, Yujie Liang^1^, Min Wang^2^, Jianchang Xu^1^, Liyan Liu^2^, Zichen Wang^1^, Gang Zeng^2^, Kelly Liu^2^, Ling Li^2,#^, Paul Yao^1,2,#^

**Supplemental Information**

**Data S1.** MATERIALS AND METHODS

**Reagents and materials**. The antibodies for β-actin (sc-47778), ERβ (sc-137381) and SOD2 (sc-30080) were obtained from Santa Cruz Biotechnology. Antibodies for H3K9me2 (ab1220), H3K9me3 (ab8898), H3K27me2 (ab24684) and H3K27me3 (ab6002), H2AX (ab20669) and γH2AX (ab2893) were obtained from Abcam, and 3-nitrotyrosine (3-NT) was measured using the 3-Nitrotyrosine ELISA Kit (ab116691 from Abcam) per manufacturers’ instructions. Protein concentration was measured using the Coomassie Protein Assay Kit (Pierce Biotechnology).

**In vivo mouse experiments.** The animal protocol conformed to US NIH guidelines (Guide for the Care and Use of Laboratory Animals, No. 85-23, revised 1996), and was reviewed and approved by the Institutional Animal Care and Use Committee from Kangning Hospital of Shenzhen. The C57Bl/6 mice were housed 4 or 5 per cage on a 12:12-h light-dark cycle and were given commercial rodent chow and water ad libitum on arrival.

Mouse Protocol 1: Generation of autistic offspring. Adult (3-month-old) female mice were monitored for estrous cycles with daily vaginal smears. Only mice with at least two regular 4- to 5-day estrous cycles were included in the studies. Chronically diabetic female mice were induced through injection of 50 mg/kg streptozocin (STZ, 0.05 M sodium citrate, pH 5.5) after an 8-hr fasting period. Animals with blood glucose >300mg/dl were considered positive, while control (CTL) mice received only vehicle injections. The females were caged with proven males, and pregnancy was verified through observation of a sperm plug, which was designated as day 0 of pregnancy. The male offspring were separated from the dams on day 21 and fed until 7-8 weeks of age for further experiments. Some of the 7-8 week-old offspring were then used for autism-like behavior testing. The amygdala was isolated for mRNA analysis, and hematopoietic stem cells (HSC) were isolated from the tibia and femur while PBMC cells were isolated from the blood for gene expression and biomedical analysis.

Mouse Protocol 2: Bone marrow transplantation (BMT) of HSC. The male offspring from CTL or STZ group in Animal Protocol 1 were used as recipients for bone marrow transplantation (BMT). HSC were harvested from the tibias and femurs of the male offspring (4 months old) that were obtained from either the CTL or STZ group in Animal Protocol 1 as the donor for BMT. The isolated HSC were purified by density centrifugation using Histopaque 1083^®^ (#-1083-1, Sigma) and then resuspended in 10ml of RPMI 1640 supplemented with 10% FBS and 2mM EDTA before being systemically transplanted (2×10^6^ cells) into the recipient male offspring (with CTL or STZ group) that had been lethally irradiated with 2 doses of 6 Gy 3 hours apart (1). All transplant-recipient mice were set aside for a minimum of 4 weeks to allow for complete reconstitution of the bone marrow (2) before they were then used for autism-like behavior analysis. PBMC were separated from blood using Ficoll-Paque Plus lymphocyte separation medium (3), and were used for analysis of gene expression and inflammatory cytokine secretion along with isolated HSC. The experimental mice were randomly separated into 4 groups as follows: CTL mouse with BMT of HSC from CTL mouse (CTL/CTL-HSC); STZ mouse with BMT of HSC from CTL mouse (STZ/CTL-HSC); CTL mouse with BMT of HSC from STZ mouse (CTL/STZ-HSC); STZ mouse with BMT of HSC from CTL mouse (STZ/CTL-HSC).

**Animal behavior test.** Animal behavior tests were carried out on offspring at 7-8 weeks of age. Autism-like behavior was evaluated using ultrasonic vocalizations, the social recognition test, and a three-chambered social test as described below (4-7).

*Ultrasonic vocalizations (USVs)*. The USVs of neonates were examined during brief maternal separation on postnatal day 7. USVs from individually-isolated pups were recorded using an externally polarized condenser microphone with a frequency range of 30-300 kHz that was attached 15-20cm above the floor of an isolation chamber. The microphone was connected to the Avisoft-UltrasoundGate recording software (Avisoft Bioacoustics, Germany) and the calls emitted from the pups were recorded to WAV sound files using parameters optimized for mice. Pups were individually placed in the sound-proof chambers and calls were recorded for 300s. Data transformation on the number of USVs were analyzed using a generalized linear model with a negative binomial distribution and a log-link function (4, 5).

*Social recognition*. Social recognition is defined by reduced time spent investigating a familiar conspecific as a result of social habituation and subsequent reinstatement of investigation when a novel intruder is introduced (dishabituation). Unfamiliar age- and sex-matched intact stimulus mice were placed in wire mesh containers. Before the test, the stimulus mice were gently habituated to being in the container and focal mice were habituated to having an empty container in their home cage. Each focal rat was tested five times (tests 1-5) in their home cage, in which a container with a stimulus rat was introduced. Each test lasted 5 min and the tests were repeated within a 15-min interval of each other. During the 15-min interval, the same empty container was placed back in the home cage of the focal rat. During the first four tests, the same stimulus rat was used, whereas for the fifth test, the stimulus rat was replaced with another unfamiliar sex- and age-matched conspecific. The placement of the containers throughout the five tests was kept constant. During the tests, the mice were left undisturbed and their behavior was videotaped and subsequently scored using JWatcher software program, with social investigation being defined as sniffing the wire mesh part of the container (5, 6).

*Three-chambered social test*. 7-8 week-old mice were used to assess sociability and preference for social novelty. Target subjects (Stranger 1 and Stranger 2) were habituated to being placed inside wire cages for 3 days prior to the beginning of testing. Test mice were habituated to the testing room for at least 45 min prior to the start of behavioral tasks. For the sociability test, the test animal was introduced to the middle chamber and left to habituate for 5 min, after which an unfamiliar mouse (Stranger 1) was introduced into a wire cage in one of the side-chambers and an empty wire cage on the other side-chamber. The test animal was allowed to freely explore all three chambers over a 10-min session. Following this time period, a novel stranger rat (Stranger 2) was introduced into the previously empty wire cage and the test animal was again left to explore for a 10-min session. Parameters scored included time spent in each chamber and number of entries into the chambers. Time spent in each chamber and track maps were calculated using automated SMART software (6).

**In vitro primary culture of amygdala neurons**. Amygdala tissues were dissected from offspring on embryonic day 18 (E18 rats). Tissues were treated with 0.05% trypsin EDTA for 15 min at 37°C. Trypsin EDTA was replaced with soybean trypsin inhibitor (Sigma) for 5 min at 37°C to stop the reaction. This was then replaced with supplemented Neurobasal A (Invitrogen) followed by mechanical dissociation. Cells were then resuspended in culture media, including Neurobasal A, B27, 1×GlutaMAX and 100 U/ml Pen/Strep (from Invitrogen), and then the cells were incubated at 37°C, 5% CO2 (8). The isolated amygdala neurons were used for analysis of epigenetic changes by ChIP assay on the SOD2 promoter (9).

**RT reaction and real-time quantitative PCR.** Total RNA from isolated cells was extracted using the RNeasy Micro Kit (Qiagen) and the RNA was reverse transcribed using an Omniscript RT kit (Qiagen). All the primers were designed using Primer 3 Plus software with the Tm at 60°C, primer size of 21bp, and the product length in the range of 140-160bp (see Table S1). The primers were validated with an amplification efficiency in the range of 1.9-2.1, and the amplified products were confirmed with agarose gel. Real-time quantitative PCR was run on iCycler iQ (Bio-Rad) with the Quantitect SYBR green PCR kit (Qiagen). PCR was performed through denaturing at 95°C for 8 min followed by 45 cycles of denaturation at 95°C, annealing at 60°C, and extension at 72°C for 10s, respectively. 1 µl of each cDNA was used to measure target genes. β-actin was used as the housekeeping gene for transcript normalization, and the mean values were used to calculate relative transcript levels with the ^ΔΔ^CT method per instructions from Qiagen. In brief, the amplified transcripts were quantified by the comparative threshold cycle method using β-actin as a normalizer. Fold changes in gene mRNA expression were calculated as 2^−ΔΔCT^ with CT = threshold cycle, ΔCT=CT (target gene)-CT(β-actin), and the ΔΔCT =ΔCT (experimental)-ΔCT (reference) (9, 10).

**Western Blotting.** Cells were lysed in an ice-cold lysis buffer (0.137M NaCl, 2mM EDTA, 10% glycerol, 1% NP-40, 20mM Tris base, pH 8.0) with protease inhibitor cocktail (Sigma). The proteins were separated in 10% SDS-PAGE and further transferred to the PVDF membrane. The membrane was blotted using primary antibodies and then incubated with the differentially labeled species-specific secondary antibodies, anti-RABBIT IRDye™ 800CW (green) and anti-MOUSE (or goat) ALEXA680 (red). Membranes were scanned and quantitated by the ODYSSEY Infrared Imaging System (LI-COR, NE) (11).

**Chromatin immunoprecipitation (ChIP).** Cells were washed and crosslinked using 1% formaldehyde for 20 min and were terminated by 0.1M glycine. Cell lysates were sonicated and centrifuged, and 500µg of protein were pre-cleared by BSA/salmon sperm DNA with preimmune IgG and a slurry of Protein A Agarose beads. Immunoprecipitations were performed with the indicated antibodies, BSA/salmon sperm DNA and a 50% slurry of Protein A agarose beads. Input and immunoprecipitates were washed, eluted, and then incubated with 0.2mg/ml Proteinase K for 2h at 42˚C, followed by 6h at 65˚C to reverse the formaldehyde crosslinking. DNA fragments were recovered through phenol/chloroform extraction and ethanol precipitation. A ~150bp fragment in the range of -300~-100 from the transcription start site on the mouse SOD2 promoter was amplified by real-time PCR (qPCR) using the primers provided in Table S1, and the amplified products were further confirmed by agarose gel (9, 10).

**Measurement of ROS generation.** Cells were seeded in a 24-well plate and incubated with 10μM CM-H2DCFDA (Invitrogen) for 45 min at 37°C, and then the intracellular formation of reactive oxygen species (ROS) was measured at excitation/emission wavelengths of 485/530nm using a FLx800 microplate fluorescence reader (Bio-Tek). The data was normalized as arbitrary units (10, 12).

**Measurement of DNA breaks**. 8-OHdG formation was measured using an OxiSelect™ Oxidative DNA Damage ELISA Kit (Cat No. STA320, from Cell Biolabs Inc.) per manufacturers’ instructions. The formation of γH2AX was measured from nuclear extracts through western blotting using H2AX as the input control (10).

**SOD2 activity assay.** SOD2 was obtained from the mitochondrial fraction that was isolated using a Pierce Mitochondria Isolation Kit (Pierce) according to manufacturers’ instructions. The successful isolation process was confirmed by the absence of TfR (transferrin receptor) protein in the mitochondria section and the absence of COXII (cytochrome c oxidase subunit II) protein in the cytosolic fraction in western blots. SOD activity was measured as described previously (13). In brief, a stable O2^.-^ source was generated through the conversion action of XOD (xanthine oxidase) from xanthine and was mixed with chemiluminescent (CL) reagents to achieve a stable light emission. The SOD2 sample injection can scavenge O2^.-^ and the subsequent decrease of chemiluminescent response is proportional to the SOD2 activity. This system can have a detection limit of 0.001U/ml within the linear range of 0.03~2.00U/ml. The results were normalized by protein concentration and expressed as Units/mg proteins (U/mg) (7, 14).

**Immunostaining**. The isolated PBMC were transferred to cover slips, and the cells were fixed in 4% paraformaldehyde for 20 min before being incubated with 0.3% Triton X-100 in PBS for 15 min. After blocking with 5% goat serum in PBS at room temperature for 30 min, cells were incubated with 8-oxo-dG anti-mouse antibody (# 4354-MC-050, from Novus Biologicals) for 12 h at 4°C and subsequently with secondary antibody Alexa Fluor 488. The cover slips were then mounted by antifade Mountant with DAPI (staining nuclei, in blue). The photographs were taken using a [Confocal Laser Microscope](https://www.sogou.com/link?url=DSOYnZeCC_qw-OVKG_MsR3KENashJ6PPMhOejy_Q5JJflCntg_rzjU2lo9-QKkufX5Qp7YP6841C08P_Gzn4lQD4cR4JDdkk5sef3Ee0PfoOX3hBKf-DUA..) (Leica, 20x lens) and quantitated by Image J. software.

**Analysis of cytokines.** Mouse cytokine secretion was obtained from PBMC supernatant, including IL-1β, IL-6 and MCP1, were measured by Mouse IL-1β/IL-1F2 Quantikine ELISA Kit (#MLB00C), Mouse IL-6 Quantikine ELISA Kit (#M6000B), and Mouse [CCL2/JE/MCP1 Quantikine ELISA Kit](https://www.rndsystems.com/products/mouse-ccl2-je-mcp-1-quantikine-elisa-kit_mje00b) (#MJE00B), respectively, according to manufacturers’ instructions from R&D Systems (15).

**Human study protocol.** The human subjects study was approved by the Human Subjects Institutional Review Board from Hainan Women and Children's Medical Center. 32 cases of ASD children and 28 cases of matched TD children (2-6 years old) were identified and subjects participated in this study with informed written consent from their parents (16). ASD diagnosis was based on several clinical assessments by a multidisciplinary team and was further confirmed by licensed clinical psychologists and psychiatrists in Hainan Women and Children's Medical Center using the DSM-5 (Diagnostic and Statisti­cal Manual of Mental Disorders, Fifth Edition) as diagnostic criteria (16-18). 3-5 ml of peripheral blood were withdrawn from the selected children and plasma was collected. Various cytokines, including IFNγ (type II interferon), IL-1α (Interleukin 1α), IL-1Rα (Interleukin 1 receptor antagonist), IL-1β, IL-6, IL-8, monocyte chemotactic protein-1 (MCP1), macrophage inflammatory protein-1α (MIP1α) and tumor necrosis factor-α (TNF-α), were measured using BIO-PLEX Pro^TM^ Human Chemokine Panel (40-Plex #171AK99MR2) according to manufacturers’ instructions from BIO-RAD. Furthermore, PBMC were isolated from fresh blood using Lymphoprep^TM^ reagents (#07861, from STEMCELL Technologies) for mRNA analysis of ERα, ERβ and SOD2. Combined PBMC from either the ASD or TD group were used for protein analysis through western blotting. The ROC (Receiver Operating Characteristic) curve was established and the Pass/Fail Cutoff Value was defined based on SOD2 mRNA levels using SPSS 22 software for screening of ASD children.

**Statistical analysis**. The data was given as mean ± SEM and all the experiments were performed at least in quadruplicate unless indicated otherwise. The unpaired Student’s t-tests or one-way analysis of variance (ANOVA) followed by the Turkey−Kramer test was used to determine statistical significance of different groups, and the two-way ANOVA followed by the Bonferroni post hoc test was used to determine the effect of social recognition. The ROC (Receiver Operating Characteristic) curve and Pass/Fail Cutoff Value was established using SPSS 22 software, and a *P* value of < 0.05 was considered significant (9, 19).

REFERENCES

1. Xie W, Ren M, Li L, Zhu Y, Chu Z, Zhu Z, Ruan Q, Lou W, Zhang H, Han Z, et al. Perinatal testosterone exposure potentiates vascular dysfunction by ERbeta suppression in endothelial progenitor cells. *PLoS One.* 2017;12(8):e0182945.

2. Ii M, Nishimura H, Iwakura A, Wecker A, Eaton E, Asahara T, and Losordo DW. Endothelial progenitor cells are rapidly recruited to myocardium and mediate protective effect of ischemic preconditioning via "imported" nitric oxide synthase activity. *Circulation.* 2005;111(9):1114-20.

3. Molloy CA, Morrow AL, Meinzen-Derr J, Schleifer K, Dienger K, Manning-Courtney P, Altaye M, and Wills-Karp M. Elevated cytokine levels in children with autism spectrum disorder. *J Neuroimmunol.* 2006;172(1-2):198-205.

4. Silverman JL, Yang M, Lord C, and Crawley JN. Behavioural phenotyping assays for mouse models of autism. *Nat Rev Neurosci.* 2010;11(7):490-502.

5. Schaafsma SM, Gagnidze K, Reyes A, Norstedt N, Mansson K, Francis K, and Pfaff DW. Sex-specific gene-environment interactions underlying ASD-like behaviors. *Proc Natl Acad Sci U S A.* 2017;114(6):1383-8.

6. Moy SS, Nadler JJ, Perez A, Barbaro RP, Johns JM, Magnuson TR, Piven J, and Crawley JN. Sociability and preference for social novelty in five inbred strains: an approach to assess autistic-like behavior in mice. *Genes Brain Behav.* 2004;3(5):287-302.

7. Wang X, Lu J, Xie W, Lu X, Liang Y, Li M, Wang Z, Huang X, Tang M, Pfaff DW, et al. Maternal diabetes induces autism-like behavior by hyperglycemia-mediated persistent oxidative stress and suppression of superoxide dismutase 2. *Proc Natl Acad Sci U S A.* 2019;116(47):23743-52.

8. Hay CW, Shanley L, Davidson S, Cowie P, Lear M, McGuffin P, Riedel G, McEwan IJ, and MacKenzie A. Functional effects of polymorphisms on glucocorticoid receptor modulation of human anxiogenic substance-P gene promoter activity in primary amygdala neurones. *Psychoneuroendocrinology.* 2014;47(43-55.

9. Zou Y, Lu Q, Zheng D, Chu Z, Liu Z, Chen H, Ruan Q, Ge X, Zhang Z, Wang X, et al. Prenatal levonorgestrel exposure induces autism-like behavior in offspring through ERbeta suppression in the amygdala. *Mol Autism.* 2017;8(46.

10. Zhang H, Li L, Li M, Huang X, Xie W, Xiang W, and Yao P. Combination of betulinic acid and chidamide inhibits acute myeloid leukemia by suppression of the HIF1alpha pathway and generation of reactive oxygen species. *Oncotarget.* 2017;8(55):94743-58.

11. Ceradini DJ, Yao D, Grogan RH, Callaghan MJ, Edelstein D, Brownlee M, and Gurtner GC. Decreasing intracellular superoxide corrects defective ischemia-induced new vessel formation in diabetic mice. *J Biol Chem.* 2008;283(16):10930-8.

12. Yao D, Shi W, Gou Y, Zhou X, Yee Aw T, Zhou Y, and Liu Z. Fatty acid-mediated intracellular iron translocation: a synergistic mechanism of oxidative injury. *Free Radic Biol Med.* 2005;39(10):1385-98.

13. Yao D, Vlessidis AG, Gou Y, Zhou X, Zhou Y, and Evmiridis NP. Chemiluminescence detection of superoxide anion release and superoxide dismutase activity: modulation effect of Pulsatilla chinensis. *Anal Bioanal Chem.* 2004;379(1):171-7.

14. Kong D, Zhan Y, Liu Z, Ding T, Li M, Yu H, Zhang L, Li H, Luo A, Zhang D, et al. SIRT1-mediated ERbeta suppression in the endothelium contributes to vascular aging. *Aging Cell.* 2016.

15. Kobayashi EH, Suzuki T, Funayama R, Nagashima T, Hayashi M, Sekine H, Tanaka N, Moriguchi T, Motohashi H, Nakayama K, et al. Nrf2 suppresses macrophage inflammatory response by blocking proinflammatory cytokine transcription. *Nat Commun.* 2016;7(11624.

16. Li L, Li M, Lu J, Ge X, Xie W, Wang Z, Li X, Li C, Wang X, Han Y, et al. Prenatal Progestin Exposure Is Associated With Autism Spectrum Disorders. *Front Psychiatry.* 2018;9(611.

17. Murphy CM, Wilson CE, Robertson DM, Ecker C, Daly EM, Hammond N, Galanopoulos A, Dud I, Murphy DG, and McAlonan GM. Autism spectrum disorder in adults: diagnosis, management, and health services development. *Neuropsychiatr Dis Treat.* 2016;12(1669-86.

18. Rausch A, Zhang W, Haak KV, Mennes M, Hermans EJ, van Oort E, van Wingen G, Beckmann CF, Buitelaar JK, and Groen WB. Altered functional connectivity of the amygdaloid input nuclei in adolescents and young adults with autism spectrum disorder: a resting state fMRI study. *Mol Autism.* 2016;7(13.

19. Glasson EJ, Bower C, Petterson B, de Klerk N, Chaney G, and Hallmayer JF. Perinatal factors and the development of autism: a population study. *Arch Gen Psychiatry.* 2004;61(6):618-27.

**Table S1. Sequences of human primers for the real time quantitative PCR (qPCR)**

| Gene | Species | Analysis | Forward primer (5'→3') | Reverse primer (5'→3') |
| --- | --- | --- | --- | --- |
| β-actin | Human | mRNA | gatgcagaaggagatcactgc | atactcctgcttgctgatcca |
| ERβ | Human | mRNA | atgatgatgtccctgaccaag | acatcagccccatcattaaca |
| ERα | Human | mRNA | gatgatgggcttactgaccaa | agacgagaccaatcatcagga |
| SOD2 | Human | mRNA | gcctacgtgaacaacctgaac | tgaggtttgtccagaaaatgc |
| SOD2 | Mouse | ChIP | aggtcactccgggcataaat | agctgcaaagcttccactcta |
| β-actin | Mouse | mRNA | tcttgggtatggaatcctgtg | atctccttctgcatcctgtca |
| ERβ | Mouse | mRNA | atgatgatgtccctgaccaag | acatcagccccatcattaaca |
| ERα | Mouse | mRNA | ccaaggagactcgctactgtg | aatggtgcattggtttgtagc |
| SOD2 | Mouse | mRNA | ggcctacgtgaacaatctcaa | tcaggtttgtccagaaaatgg |

FIGURE S1

**Figure S1. Representative pictures of full blots for Western Blotting.** (a) Representative full blots for Figure 1i. (b) Representative full blots for Figure 2f. (c). Representative full blots for Figure 3e. (d). Representative full blots for Figure 5c.

FIGURE S2

**Figure S2.** **Transplantation of bone marrow HSC does not restore maternal diabetes-induced autism-like behavior in offspring.** The 6-week old male offspring from either the control (CTL) or maternal diabetes (STZ) group received transplantation of bone marrow HSC from either the control (CTL-HSC) or maternal diabetes (STZ-HSC) group. Five weeks after transplantation, the mice were used for autism-like behavior analysis and the amygdala tissues and neurons were isolated for analysis. (a) ChIP analysis on the SOD2 promoter in amygdala neurons, n=4. (b) mRNA levels in the amygdala, n=5. (c) Ultrasonic vocalization, n=9. (d) Social recognition, seconds spent socially investigating a conspecific [same conspecific in tests 1-4; novel conspecific in test 5 (a new stimulus mouse was introduced)], n=9. (e,f) Three-chambered social tests, n=8. (e) Time spent in chamber, indicating sociability. (f) Time spent in chamber, indicating social novelty. *, *P*<0.05, vs CTL group. Data were expressed as mean ± SEM.

FIGURE S3

**Figure S3.** **Establishment of** **Cut/Off value for the diagnosis of ASD patients based on SDO2 mRNA expression**. (a) The coordinates of the curve, part a. (b) The coordinates of the curve, part b. (c) The coordinates of the curve, part c.
